# Resistance of *Arabidopsis thaliana* to the green peach aphid, *Myzus persicae*, involves camalexin and is regulated by microRNAs

**DOI:** 10.1111/nph.12218

**Published:** 2013-03-25

**Authors:** Graeme J Kettles, Claire Drurey, Henk-jan Schoonbeek, Andy J Maule, Saskia A Hogenhout

**Affiliations:** 1 Department of Cell and Developmental BiologyNorwich Research Park, Norwich, Norfolk, NR4 7UH, UK; 2 Department of Crop Genetics, John Innes CentreNorwich Research Park, Norwich, Norfolk, NR4 7UH, UK

**Keywords:** aphid resistance, camalexin, glucosinolates, microRNA, *PAD3*

## Abstract

- Small RNAs play important roles in resistance to plant viruses and the complex responses against pathogens and leaf-chewing insects. We investigated whether small RNA pathways are involved in *Arabidopsis* resistance against a phloem-feeding insect, the green peach aphid (*Myzus persicae*).
- We used a 2-wk fecundity assay to assess aphid performance on *Arabidopsis* RNA silencing and defence pathway mutants. Quantitative real-time polymerase chain reaction was used to monitor the transcriptional activity of defence-related genes in plants of varying aphid susceptibility. High-performance liquid chromatography-mass spectrometry was employed to measure the accumulation of the antimicrobial compound camalexin. Artificial diet assays allowed the assessment of the effect of camalexin on aphid performance.
- *Myzus persicae* produces significantly less progeny on *Arabidopsis* microRNA (miRNA) pathway mutants. Plants unable to process miRNAs respond to aphid infestation with increased induction of *PHYTOALEXIN DEFICIENT3* (*PAD3*) and production of camalexin. Aphids ingest camalexin when feeding on *Arabidopsis* and are more successful on *pad3* and *cyp79b2*/*cyp79b3* mutants defective in camalexin production. Aphids produce less progeny on artificial diets containing camalexin.
- Our data indicate that camalexin functions beyond antimicrobial defence to also include hemipteran insects. This work also highlights the extensive role of the miRNA-mediated regulation of secondary metabolic defence pathways with relevance to resistance against a hemipteran pest.

## Introduction

The green peach aphid (GPA), *Myzus persicae*, is one of the most destructive pests on cultivated crops worldwide (Blackman & Eastop, 2000). GPA causes feeding damage and, more importantly, is the vector of many different plant viruses (Ng & Perry, 2004; Hogenhout *et al*., 2008). Insect herbivores, including aphids, have often specialized to colonize one or a few related plant species, whereas only a few herbivores, such as GPA, can colonize diverse plant species. Therefore, most plants can defend themselves effectively against the majority of insect herbivores. Moreover, insects are probably required to modulate a variety of plant processes to facilitate colonization. However, the mechanisms by which plants defend themselves against insect colonization and how aphids modulate plant processes are not fully understood.

Aphids possess specialized mouthparts, named stylets, which are developed for the piercing of plant tissues and the ingestion of sap, and allow them to feed from phloem tissue (Tjallingii, 2006). Access to this tissue is gained following extensive probing by the stylets of epidermal and parenchymal cell layers, before the establishment of a successful feeding site in the phloem sieve element (Tjallingii & Esch, 1993). Once established, feeding can be maintained for several hours (Tjallingii, 1995).

In plants, small RNAs (sRNAs) regulate changes in gene expression in response to a variety of biotic and abiotic stimuli (Sunkar & Zhu, 2004; Fujii *et al*., 2005; Ruiz-Ferrer & Voinnet, 2009; Katiyar-Agarwal & Jin, 2010). It has long been known that components of sRNA pathways play an extensive role in antiviral defence (Ding & Voinnet, 2007). More recently, sRNA pathways have been implicated in resistance to bacteria, fungi, nematodes and insects (Navarro *et al*., 2006; Pandey & Baldwin, 2007; Hewezi *et al*., 2008; Pandey *et al*., 2008; Ellendorff *et al*., 2009). sRNAs modify gene expression by acting at both the transcriptional and post-transcriptional levels (Voinnet, 2009). RNA-induced silencing is initiated by double-stranded RNA (dsRNA), which can occur as a stem-loop precursor, or a longer dsRNA molecule generated by either bidirectional transcription or the action of an RNA-dependent RNA polymerase (RDR) on a single-stranded RNA (ssRNA) template (Ruiz-Ferrer & Voinnet, 2009). In Arabidopsis, segments of dsRNA are cleaved into 18–24-nucleotide (nt) sRNA duplexes by one or a combination of four Dicer-like (DCL) endoribonucleases. Following methylation of the 2-nt 3′ overhang by the methyltransferase HUA ENHANCER1 (HEN1; Yu *et al*., 2005), sRNA can be exported from the nucleus before incorporation into an RNA-induced silencing complex (RISC) containing one of 10 Argonaute (AGO) proteins (Vazquez *et al*., 2010). The sRNA guides the RISC to either cleave or repress the translation of target transcripts bearing sufficient homology to the loaded sRNA.

sRNAs can be divided into subgroups depending on their source and mode of processing (Vazquez *et al*., 2010). Small interfering RNA (siRNA) is processed from segments of long, perfectly complementary dsRNA, which may be derived from pathogens (e.g. viruses) or generated from loci throughout the genome, but especially from highly repetitive regions (Rabinowicz *et al*., 2003; Matzke *et al*., 2007). The latter is consistent with the known role for siRNAs in directing heterochromatic silencing of genomic regions harbouring mobile genetic elements (Matzke *et al*., 2007). MicroRNAs (miRNAs) are a class of largely 21-nt sRNAs derived from imperfectly complementary stem-loop precursors. miRNAs are excised from their precursors by DCL1 (Park *et al*., 2002; Kurihara & Watanabe, 2004), although the rate and fidelity of this excision is dependent on the cofactors SERRATE (SE) and HYPONASTIC LEAVES 1 (HYL1; Dong *et al*., 2008). miRNAs are subject to methylation by HEN1 and are exported from the nucleus via both HASTY (HST)-dependent and independent mechanisms (Park *et al*., 2005). At some point, there is unravelling of the duplex into its component miR and complementary miR* strands, before one strand is selectively incorporated into RISC. AGO1 is the dominant slicer of the miRNA pathway (Baumberger & Baulcombe, 2005), although a proportion is reported to act through AGO7 or AGO10 (Brodersen *et al*., 2008; Montgomery *et al*., 2008).

The miRNA pathway is known to play a significant role in the regulation of the defence response that occurs following challenge by the bacterial biotroph *Pseudomonas syringae* (Navarro *et al*., 2006; Zhang *et al*., 2011) and the pathogen-associated molecular pattern (PAMP) flg22 (Li *et al*., 2010). The defence pathways activated in response to attack from chewing herbivores are also governed by sRNAs. The growth of *Manduca sexta* (tobacco hornworm) larvae is enhanced on *Nicotiana attenuata* lacking RDR1 (Pandey & Baldwin, 2007). In this interaction, RDR1-dependent siRNAs are required to coordinate a defence response involving nicotine biosynthesis and the jasmonic acid (JA) and ethylene (ET) signalling pathways (Pandey *et al*., 2008).

Aphid infestations elicit transcriptional reprogramming in host plants, despite causing little visible feeding damage (Moran *et al*., 2002; Couldridge *et al*., 2007; Kusnierczyk *et al*., 2007, 2008; Gao *et al*., 2010). In one study, these changes were more pronounced than those elicited by fungal or bacterial pathogens, or a leaf-chewing lepidopteran pest (De Vos *et al*., 2005). miRNAs, in particular, are known to target large families of transcription factors. Infestation by several aphid species also results in large-scale changes in the transcription factor profile of infested tissue (Kusnierczyk *et al*., 2008; Gao *et al*., 2010; Sattar *et al*., 2012). Given these observations and the known involvement of sRNAs in defence responses against pathogens and a chewing herbivore, we speculated that sRNAs may play a similarly important role in coordinating the complex and large-scale response to aphids.

GPA effectively colonizes members of the family Brassicaceae, including the model plant *Arabidopsis thaliana*. Here, we report that Arabidopsis plants deficient in miRNA processing show increased resistance to GPA. This resistance is partly a result of the enhanced production of the phytoalexin camalexin, which is known to play a role in plant defence against bacterial and fungal microbial pathogens. Camalexin is produced at GPA stylet penetration sites, and this plant compound accumulates in aphids fed on plants and an artificial diet containing camalexin. Progeny production is reduced in aphids exposed to camalexin, whereas aphids produce more progeny on plants compromised in camalexin production. Together, this work uncovers a novel role for camalexin in modifying insect reproductive ability.

## Materials and Methods

### Aphids

Stock colonies of *M. persicae* (Sulzer) (RRes genotype O; GPA; Bos *et al*., 2010) were reared in 52 × 52 × 50-cm^3^ cages containing up to six Chinese cabbage (*Brassica rapa*, subspecies *chinensis*) plants with a 14-h day (90 μmol m^−2^ s^−1^ at 18°C) and a 10-h night (15°C) cycle.

### Plant growth conditions

All plants used in this investigation belong to the Arabidopsis Col-0 ecotype. The *ago1-25*, *ago1-26* and *ago1-27* mutants (Morel *et al*., 2002) were supplied by Hervé Vaucheret (Laboratoire de Biologie Cellulaire, INRA Centre de Versailles, Versailles Cedex, France). The *dcl1-9*, *hen1-5*, *rdr1-1*, *rdr2-1* and *rdr6* mutants (Jacobsen *et al*., 1999; Mourrain *et al*., 2000; Vazquez *et al*., 2004b; Xie *et al*., 2004) were kindly provided by Fuquan Liu (Queen's University, Belfast, UK). The *dcl2*, *dcl3*, *dcl4*, *dcl2*/*3*, *dcl2*/*4* and *dcl2*/*3*/*4* mutants (Xie *et al*., 2004, 2005; Henderson *et al*., 2006) were obtained from Olivier Voinnet (Swiss Federal Institute of Technology, Zurich, Switzerland). The *hst*, *se1*, *ago2*, *ago4*, *ago7*, *cyp81f2*, 35S:LOX2 and 35S:LOX2 antisense lines (Bell *et al*., 1995; Bollman *et al*., 2003; Zilberman *et al*., 2003; Vazquez *et al*., 2004a; Lobbes *et al*., 2006; Pfalz *et al*., 2009) were provided by the Nottingham Arabidopsis Stock Centre (NASC, Nottingham, UK). *dcl1.fwf2* and *fwf2* (Katiyar-Agarwal *et al*., 2007) were kindly provided by Rebecca Mosher (University of Arizona, Tucson, AZ, USA). The *phytoalexin deficient3* (*pad3*), *nonexpressor of pathogenesis-related genes1* (*npr1*) and *salicylic acid induction-deficient2* (*sid2*) mutants (Cao *et al*., 1994; Glazebrook & Ausubel, 1994; Nawrath & Metraux, 1999) were obtained from Alexandre Robert-Seilaniantz (Sainsbury Laboratory, Norwich, Norfolk, UK). The *cyp79b2*/*cyp79b3* double mutant (Zhao *et al*., 2002) was obtained from Jean-Pierre Métraux (University of Fribourg, Fribourg, Switzerland). The *coronatine insensitive1* (*coi1-35*) and *jasmonate resistant1* (*jar1*) mutants (Staswick *et al*., 1992) were provided by Jonathan Jones (Sainsbury Laboratory). The *ethylene insensitive2* (*ein2-5*) and *ethylene resistant1* (*etr1-1*) mutants (Bleecker *et al*., 1988; Alonso *et al*., 1999) were from Freddy Boutrot (Sainsbury Laboratory). The *CYP71B15p::GUS* (*PAD3p::GUS*) transgenic lines (Schuhegger *et al*., 2006) were supplied by Erich Glawischnig (Technische Universität München, Munich, Germany).

All Arabidopsis plants used in the aphid fecundity experiments were germinated and maintained on Scotts Levington F2 compost. Seeds of the Arabidopsis sRNA mutants were vernalized at 4°C for 72 h and grown in a controlled environment room (CER) with an 8-h day (90 μmol m^−2^ s^−1^ at 18°C) and 16-h night (16°C) cycle. Two-week-old seedlings were transferred to seedling trays containing 24 modules. Plants were used for experiments after a further 2 wk when they were 4 wk old.

Seeds of the Arabidopsis hormone/secondary metabolite pathway mutants were vernalized for 1 wk at 5–6°C and grown in a CER with a 10-h day (90 μmol m^−2^ s^−1^ at 22°C) and a 14-h night (22°C) cycle. Plants were used for experiments at 4 wk old.

### Aphid fecundity assays

All fecundity assays were carried out in a CER with an 8-h day (90 μmol m^−2^ s^−1^ at 18°C) and a 16-h night (16°C) cycle. Four-week-old plants were potted into 1-l round black pots (diameter, 13 cm; height, 10 cm) containing fresh compost, and were caged in clear plastic tubing (diameter, 10 cm; height, 15 cm; Jetran tubing; Bell Packaging Ltd, Luton, UK) capped at the top with white gauze-covered plastic lids. Each plant was seeded with four adult GPA from the stock colony, and the plants were returned to the CER. After 48 h, all adults were removed from the test plants (day 0) and the plants were returned to the growth room. On day 3, excess nymphs were removed, leaving five nymphs per plant. On day 11, when most nymphs had reached adulthood and started to produce their own offspring, the numbers of these new nymphs were counted. The newly produced nymphs were removed and the adults remained on the plant. On day 14, a second nymph count was carried out, together with a count of the surviving adults. Experiments were terminated on day 14. The total number of nymphs produced was calculated by combining the day 11 and day 14 nymph counts. Each experiment included five plants per genotype that were arranged in trays using a randomized block design, and each experiment was repeated at least twice. The experiment to assess aphid performance over a shorter period was performed following a method described previously (Pegadaraju *et al*., 2005).

All statistical analyses were conducted using the GenStat 11 statistical package (VSNi Ltd, Hemel Hempstead, Hertfordshire, UK). Data were checked for approximate normal distribution by visualizing residuals. Classical linear regression analysis using a generalized linear model (GLM) with Poisson distributions was applied to analyse the GPA fecundity on plants with ‘nymphs’ as a response variable. The aphid nymph production on five plants per genotype was used as an independent data point in statistical analyses in which the biological replicate was used as a variable.

### Single-leaf aphid infestations

Thirty GPA nymphs from the stock cage were transferred to a single clip-cage and confined to a single mature rosette leaf of a 5-wk-old plant at one clip-cage per plant. Plants were returned to the CER for the appropriate infestation period. Two to four aphid-exposed leaves per treatment were pooled to produce each sample, and the leaves caged with aphid-free clip-cages were used as controls. For the 12-h infestations of the RNA silencing mutants, three independent experiments were conducted containing three, four and two biological replicates, respectively. This gave nine biological replicates in total, which were statistically analysed together. The 24- and 48-h infestations of the RNA silencing mutants contained four biological replicates. For the 6-, 12-, 24- and 48-h Col-0 infestation time courses, four biological replicates of each treatment were analysed.

### Quantitative real-time polymerase chain reaction (qRT-PCR)

Pooled leaf samples were ground in chilled 1.5-ml Eppendorf tubes using disposable pellet pestles (Sigma-Aldrich, St Louis, MO, USA). Total RNA was extracted using Tri-Reagent (Sigma-Aldrich) and included a DNaseI treatment (RQ1 DNase set; Promega, Madison, WI, USA). RNA was purified using the RNA cleanup protocol of the RNeasy Mini Kit (Qiagen, Hilden, Germany). cDNA was synthesized from 500 ng RNA using the MMLV-RT Kit (Invitrogen, Carlsbad, CA, USA) and oligo dT primer, following the manufacturer's instructions. cDNA from these reactions was diluted 1 : 20 with distilled H_2_O before qRT-PCR.

Twenty-microlitre reactions were set up in 96-well white ABgene PCR plates (Thermo Scientific, Loughborough, Leicestershire, UK) in a CFX96 Real-Time System with a C1000 Thermal Cycler (Bio-Rad, Hemel Hempstead, Hertfordshire, UK) using SYBR Green JumpStart Taq ReadyMix (Sigma-Aldrich).

All reactions were carried out using the following thermocycle: 3 min at 95°C, followed by 40 cycles of (30 s at 95°C, 30 s at 60°C, 30 s at 72°C), followed by melt curve analysis: 30 s at 50°C (65–95°C at 0.5°C increments, 5 s for each).

Reference genes for this study were chosen from a selection of candidates previously identified as superior reference genes (Czechowski *et al*., 2005). Using geNORM (Vandesompele *et al*., 2002), it was established that ACT2 (At3g18780), Clathrin adapter complex subunit (At5g46630) and PEX4 (At5g25760) were the most stable across a range of mock and GPA-exposed Arabidopsis rosette leaf RNA samples. Mean *C*_t_ values for each sample–primer pair combination were calculated from two or three replicate reaction wells. Mean *C*_t_ values were then converted to relative expression values using the formula 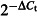. The geometric mean of the relative expression values of the three reference genes was calculated to produce a normalization factor unique to each sample. Relative expression values for each gene of interest were normalized using the normalization factor for each sample. The normalized expression values for each gene of interest were then compared between mock and aphid-exposed samples across all plant lines tested in the experiment. Analysis of variance (ANOVA) was performed to assign variance attributable to plant genotype, block and replicate using a GLM in GenStat. Means were compared by calculating *t* probabilities within the GLM. Primer sequences for both reference and target genes are available in Supporting Information Table S1.

### Camalexin extraction and measurement

For plant samples, single leaves from 5-wk-old Arabidopsis were infested with 30 GPA nymphs and the leaves were confined with clip-cages. Leaves treated with empty clip-cages were used as controls. Both mock and aphid-infested leaves were harvested after 48 h. Camalexin extractions were carried out using a method based on work described previously (Meuwly & Metraux, 1993). Samples were analysed by high-performance liquid chromatography (HPLC) on a Surveyor instrument (Thermo Scientific) attached to a DecaXP^plus^ ion trap mass spectrometer (Thermo Scientific). Camalexin and *o-*anisic acid were separated on a Luna C18(2) column (50 mm × 2 mm, 3 μm; Phenomenex, Macclesfield, UK). All peak areas were integrated using the Xcalibur software Genesis algorithm (Thermo Scientific). Each experiment contained three biological replicates of each genotype–treatment combination and the experiment was conducted twice.

For camalexin measurements in aphids, 120 nymphs were used to infest whole 5-wk-old Arabidopsis plants. After 48 h, aphids were harvested and camalexin was extracted using the same protocol as described for plant samples. Each experiment contained three biological replicates of each treatment and the experiment was conducted twice.

### Artificial diet experiments

Aphid feeders were constructed by cutting the top 2-cm portion of a 50-ml Corning tube and reattaching the lid. Parafilm was stretched over the open end to form a feeding sachet containing 100 μl of artificial diet. We used an artificial diet previously described for these experiments (Kim & Jander, 2007). Aphids were fed diet alone, dimethylsulfoxide (DMSO)-spiked (0.1%) diet or diet containing the indicated concentration of camalexin. Synthetic camalexin was provided by Jean-Pierre Métraux (University of Fribourg, Fribourg, Switzerland; Stefanato *et al*., 2009). Ten adult aphids from the stock cage were added to each feeder. Feeders were inverted, covered with a yellow plastic sheet and placed in a CER with an 8-h day (90 μmol m^−2^ s^−1^ at 18°C) and 16-h night (16°C) cycle. The number of surviving adults (from 10) and the number of nymphs produced were assessed after 48 h. Each experiment contained five feeders per treatment and the experiment was conducted twice. ANOVA was performed to assign variance attributable to diet treatment and replicate using a GLM in GenStat. Means were compared by calculating *t* probabilities within the GLM.

### β-Glucuronidase (GUS) staining

Leaves of 4-wk-old transgenic Arabidopsis lines expressing *CYP71B15p::GUS* (*PAD3p::GUS*) were infested with 30 GPA nymphs contained within clip-cages. Leaves with empty clip-cages were used as negative controls and leaves treated with *Botrytis cinerea* (B05.10) were used as positive controls. After 48 h, aphids were carefully removed and leaves were immediately submerged in GUS staining solution (0.2 M Na_2_HPO_4_, 0.2 M NaH_2_PO_4_·2H_2_O, 10% Triton X-100, 10 mM EDTA, pH 7) containing 50 mg ml^−1^ X-Gluc (5-bromo-4-chloro-3-indolyl-β-d-glucuronic acid) and 0.3% H_2_O_2_. Leaves were vacuum infiltrated with staining solution and returned to normal atmospheric pressure. This was repeated three times. Leaves were incubated in staining solution for 16 h at 37°C in the dark before destaining in 70% ethanol. Leaves were mounted on glass microscope slides in 40% glycerol and viewed under a Nikon Eclipse 800 light microscope (Nikon UK Ltd) attached to a Pixera Pro ES600 digital camera (Pixera UK Ltd).

## Results

### Aphid fecundity is reduced on Arabidopsis miRNA mutants

**Fig. 1:**
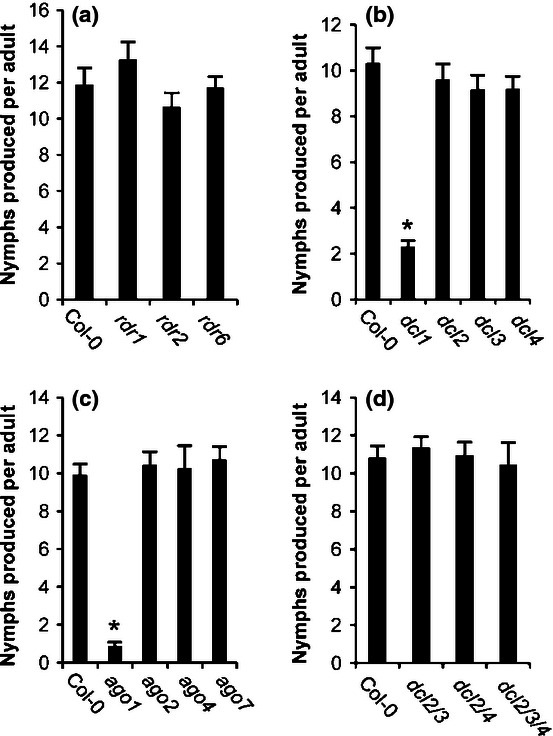
The *Arabidopsis* microRNA (miRNA) pathway is involved in aphid resistance. Aphid fecundity is reduced on miRNA pathway mutants (*dcl1*, *ago1*) (b, c), but not on mutants in other small interfering RNA (siRNA) pathways (a–d). Each plant was seeded with five nymphs, and the average fecundity of these nymphs as they progressed to adulthood was recorded. Bars represent the mean (± SE) of five plants of each genotype. Each experiment was repeated at least twice with similar results. Asterisks represent *P* < 0.001 as determined by analysis of deviance (ANODE; GenStat).

To determine whether sRNAs are involved in Arabidopsis resistance to GPA, aphid performance was assessed on a collection of RDR, DCL and AGO mutants and wild-type Col-0 Arabidopsis. In our assay, 4-wk-old plants were seeded with five nymphs aged < 48 h. These nymphs were allowed 14 d to develop to adulthood and to produce offspring. The number of offspring produced was recorded as fecundity. In our initial experiment, fecundity was unchanged among three RDR mutants (*rdr1*, *rdr2*, *rdr6*) compared with Col-0 (Fig. 1a). This indicates that RDRs are not involved in *Arabidopsis* resistance to GPA, unlike the *rdr1* mutant of *N. attenuate*, which shows decreased resistance to the herbivore *Manduca sexta* (Pandey & Baldwin, 2007). By contrast, aphids produced significantly fewer offspring on *dcl1* mutants relative to Col-0 (*t* probabilities within GLM, *P* < 0.001, *n* = 5), but were not affected on *dcl2*, *dcl3* or *dcl4* mutants (Fig. 1b). In addition, aphid fecundity was significantly lower on the *ago1-25* mutant (GLM, *P* < 0.001, *n* = 5), but was unchanged on *ago2*, *ago4* and *ago7* mutants (Fig. 1c). Aphid performance was also not affected on the *dcl2*/*3* and *dcl2*/*4* double mutants or the *dcl2*/*3*/*4* triple mutant (Fig. 1d). Because DCL1 and AGO1 both process sRNAs in the miRNA pathway, these data suggest that the miRNA pathway is involved in Arabidopsis resistance to GPA, whereas other sRNA processing pathways do not appear to play a significant role.

**Fig. 2:**
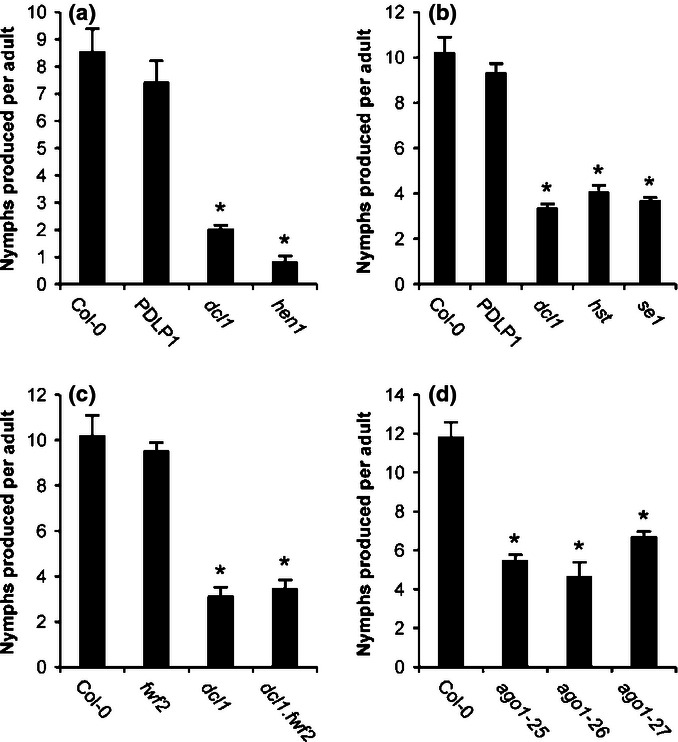
Plant stature has no effect on aphid fecundity. Aphid fecundity is reduced on Arabidopsis lines that aberrantly process microRNA (miRNA) (*hen1*, *hst*, *se1*), but remains high on the unrelated dwarf Plasmodesmata Located Protein 1 (PDLP1) line (a, b). Reduced fecundity is also observed on the partial *dcl1* rescue line *dcl1.fwf2* (c) and across several *ago1* alleles (d). Bars represent the mean (± SE) of five plants of each genotype. Each experiment was repeated at least twice with similar results. Asterisks represent *P* < 0.001 as determined by analysis of deviance (ANODE).

To investigate this further, we conducted GPA fecundity assays on other mutants in the miRNA pathway. In addition, to determine whether the smaller stature of *dcl1* and *ago1* mutants affects aphid fecundity, we included the Arabidopsis Plasmodesmata Located Protein 1 (PDLP1) overexpression line 35S::PDLP1a:GFP (Thomas *et al*., 2008) as a control, as this line exhibits a dwarfing phenotype similar to the miRNA mutants (Fig. S1). We observed that aphid fecundity was not significantly different between PDLP1 and Col-0, whereas aphids produced significantly fewer nymphs on the miRNA mutant *dcl1* and the *hen1* mutant, which is deficient in all sRNA pathways (GLM, *P* < 0.001, *n* = 5; Fig. 2a). Similarly, aphids were significantly less fecund on *hst* and *se* mutants compared with both Col-0 and PDLP1 (GLM, *P* < 0.001, *n* = 5; Fig. 2b). SE is a zinc finger protein that assists DCL1 in the accurate excision of miRNAs from their precursors, and HST is involved in the export of miRNAs from the nucleus (Park *et al*., 2005; Dong *et al*., 2008). To provide additional evidence that plant stature does not affect aphid fecundity, we also assessed aphid performance on the partial *dcl1* rescue line *dcl1.fwf2*, which retains impaired miRNA processing, but exhibits a less dwarf phenotype (Katiyar-Agarwal *et al*., 2007; Fig. S1). Fecundity on these plants matched that of *dcl1*-raised aphids (Fig. 2c). We also obtained other *ago1* alleles reported to have various degrees of dwarfism (Morel *et al*., 2002). Aphid fecundity was comparable across all of these lines (Fig. 2d), although, in our growth conditions, the *ago1-26* and *ago1-27* mutants were similar in size and stature to the *ago1-25* mutant analysed in Fig. 1(c) (Fig. S1). Nonetheless, these results suggest that the miRNA pathway is involved in the regulation of the plant resistance response to GPA, whereas other siRNA pathways are not involved. Furthermore, the resistance exhibited by miRNA pathway mutants is independent of the dwarfism phenotype.

### Camalexin, ET and JA pathway transcripts are upregulated in aphid-exposed *dcl1* mutants

Arabidopsis responses to aphid attack have been investigated extensively and involve the salicylic acid (SA), JA, ET, glucosinolate and camalexin pathways (Moran *et al*., 2002; De Vos *et al*., 2005; Couldridge *et al*., 2007; Kusnierczyk *et al*., 2007, 2008). We investigated whether the induction of these pathways was altered in an miRNA mutant by comparing the expression levels of a range of marker genes illustrative of these pathways by qRT-PCR. To assess the temporal aspect of the response, we measured defence induction in Col-0 at 6, 12, 24 and 48 h post-inoculation (hpi), and found that defence gene inductions were first reliably detected at 12 hpi, and were higher and did not change dramatically between the 24- and 48-hpi time points (Fig. S2). Therefore, we selected the 12-hpi time point as it would be possible to detect a decrease as well as an increase in gene expression levels.

**Fig. 3:**
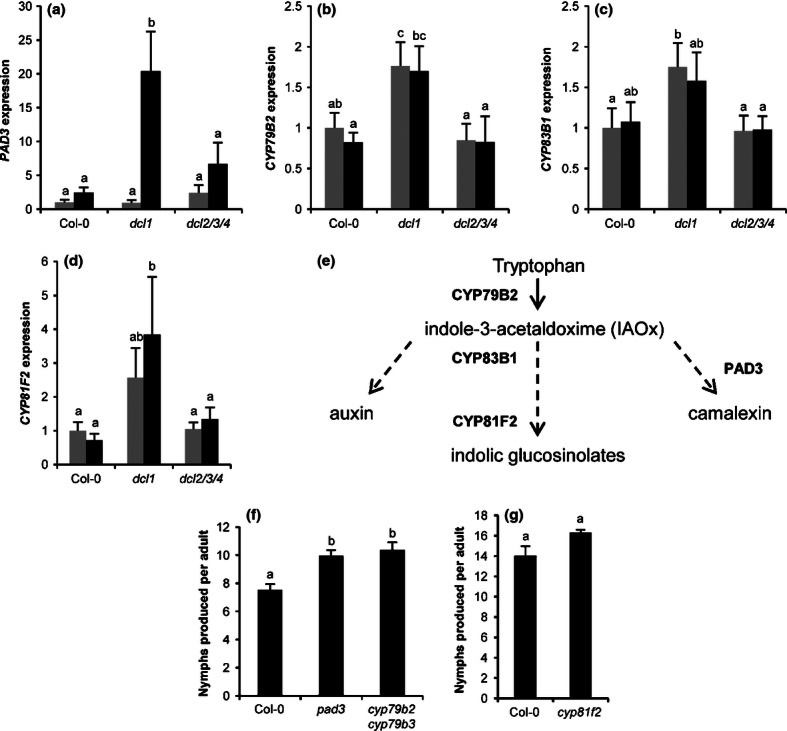
MicroRNA (miRNA) mutants show differential expression of enzymes involved in tryptophan-derived secondary metabolism. Quantitative real-time polymerase chain reaction (qRT-PCR) analysis of transcripts involved in the production of (a) camalexin (*PAD3*), (b) camalexin/indole glucosinolates (*CYP79B2*) and (c, d) indole glucosinolates (*CYP83B1*, *CYP81F2*) following 12 h of aphid infestation. miRNA mutants (*dcl1*) show greater induction of *PAD3* and *CYP81F2* relative to Col-0 and *dcl2*/*3*/*4*, and also show increased basal expression of *CYP79B2* and *CYP83B1*. Mock, grey bars; aphids, black bars. Bars represent the mean expression levels (± SE) across nine biological replicates from three independent experiments. Letters indicate differences at *P* < 0.05 as determined by *t* probabilities within a generalized linear model (GLM). (e) Position of *PAD3*, CYP79B2, CYP83B1 and CYP81F2 in the camalexin and indole glucosinolate biosynthetic pathways. (f) Aphid fecundity is similarly increased on camalexin-deficient (*pad3*) and camalexin/indole glucosinolate-deficient (*cyp79b2*/*cyp79b3*) mutants, indicating that camalexin production is the major resistance factor. (g) Aphid fecundity is unchanged on *cyp81f2* mutants. Bars represent the mean (± SE) of 10 plants of each genotype from two experiments. Letters indicate differences at *P* < 0.05 as determined by analysis of deviance (ANODE).

**Fig. 4:**
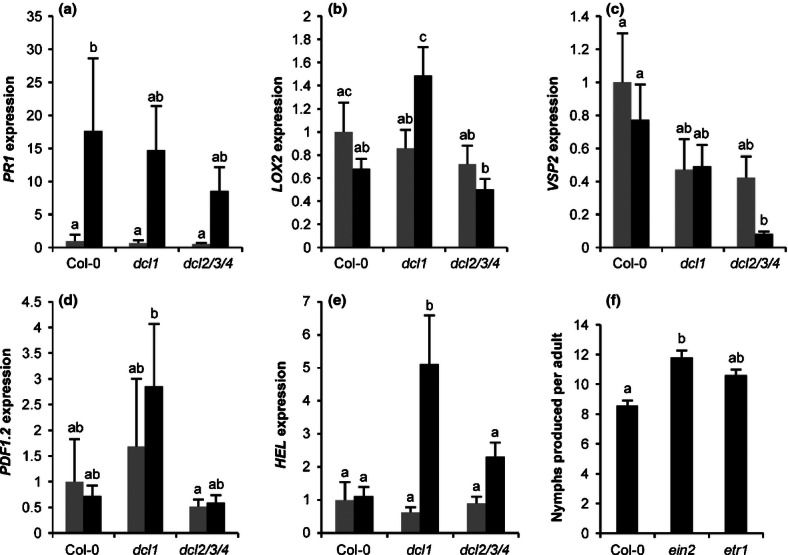
MicroRNA (miRNA) mutants show altered expression of genes involved in jasmonic acid (JA) synthesis and ethylene (ET) response. Quantitative real-time polymerase chain reaction (qRT-PCR) analysis of transcripts involved in (a) salicylic acid (SA; *PR1*), (b, c) JA (*LOX2*, *VSP2*), (d) JA/ET (*PDF1.2*) and (e) ET (*HEL*) pathways following 12 h of aphid infestation. The expression of *LOX2* and *HEL* was increased in *dcl1* relative to both Col-0 and *dcl2*/*3*/*4*. Bars represent the mean expression levels (± SE) across nine biological replicates from three independent experiments. Letters indicate differences at *P* < 0.05 as determined by *t* probabilities within a generalized linear model (GLM). Mock, grey bars; aphids, black bars. (f) Aphid fecundity is increased on *ethylene-insensitive2* (*ein2*) mutants. Bars represent the mean (± SE) of 10 plants of each genotype from two experiments. The experiment was repeated with similar results. Letters indicate differences at *P* < 0.05 as determined by analysis of deviance (ANODE).

*PAD3* (*CYP71B15*), a marker for the camalexin biosynthetic pathway (Chassot *et al*., 2008; Xu *et al*., 2008), was most strikingly induced on exposure to aphids in the *dcl1* mutant compared with Col-0 and the *dcl2*/*3*/*4* triple mutant among all the genes tested (Figs 3a, S4). In addition, *CYP81F2*, a gene involved in the indolic glucosinolate pathway, was induced significantly in aphid-infested *dcl1* plants compared with Col-0 and *dcl2*/*3*/*4* (Fig. 3d). The JA biosynthetic gene *LIPOXYGENASE2* (*LOX2*) was also upregulated significantly in aphid-exposed *dcl1* compared with aphid-exposed Col-0 and *dcl2*/*3*/*4* (Fig. 4b). The defence-related gene *MITOGEN-ACTIVATED PROTEIN KINASE3* (*MPK3*) was most strongly induced in *dcl1*, although the increase was not significantly different from aphid-exposed Col-0 or *dcl2*/*3*/*4* (Fig. S3). *PATHOGENESIS-RELATED1* (*PR1*), which has been used as a marker for SA signalling (De Vos *et al*., 2005; Kusnierczyk *et al*., 2007), is upregulated on aphid exposure; however, its induction was not significantly different among the Col-0, *dcl1* and *dcl2*/*3*/*4* plants (Fig. 4a). The basal expression levels of some genes, such as *CYP79B2* and *CYP83B1* of the indole glucosinolate/camalexin pathways, were greater in *dcl1* compared with Col-0 and *dcl2*/*3*/*4*, but did not alter significantly in any line on exposure to aphids (Fig. 3b,c). *VEGETATIVE STORAGE PROTEIN2* (*VSP2*) and *PLANT DEFENSIN1.2* (*PDF1.2*) have been used as downstream markers of the JA and ET pathways (De Vos *et al*., 2005). We found that the expression of these genes was either stable or repressed following aphid treatment, and did not differ significantly across any of the lines tested (Fig. 4c,d). By contrast, the ET-responsive transcript *HEVEIN-LIKE* (*HEL*) (*PR4*) was induced significantly in aphid-exposed *dcl1* plants compared with aphid-exposed Col-0 and *dcl2*/*3*/*4* (Figs 4e, S4). As genes involved in glucosinolate and camalexin biosynthesis and the JA and ET signalling pathways were differentially regulated in *dcl1* plants, we predicted that these pathways may be responsible for the aphid-resistant phenotype exhibited by Arabidopsis miRNA pathway mutants.

### GPA fecundity is increased on camalexin-deficient plants

The cytochrome P450 *PAD*3 catalyses the conversion of dihydrocamalexic acid to camalexin, the major Arabidopsis phytoalexin (Schuhegger *et al*., 2006; Fig. 3e). CYP81F2 is involved in a downstream part of the indolic glucosinolate pathway that has been shown to have relevance to aphid resistance (Pfalz *et al*., 2009; Fig. 3e). To investigate the contribution of *PAD*3, CYP81F2 and CYP79B2/CYP79B3 (which act upstream of the glucosinolate and camalexin pathways), we exposed the *pad3* (camalexin-deficient), *cyp81f2* (aphid-relevant glucosinolate-deficient) and *cyp79b2*/*cyp79b3* (camalexin and indole glucosinolate-deficient) mutants to insects. Aphid fecundity was significantly higher on both *pad3* and *cyp79b2*/*cyp79b3* mutants compared with Col-0 (GLM, *P* < 0.05*, n* = 10; Fig. 3f). However, aphid fecundity was not significantly different on *cyp79b2*/*cyp79b3* plants compared with *pad3*. It is possible that the aphid reproduction activity is maximized on each of the mutant plants to the degree that the absence of both camalexin and indole glucosinolates adds relatively little to aphid reproduction. Nonetheless, this indicates that the blocking of the camalexin pathway has a negative effect on aphid reproduction. The increased aphid performance on *pad3* mutants agreed with the finding that *PAD3* expression was highly induced in aphid-resistant *dcl1* plants. We found that aphid fecundity was increased on the *cyp81f2* mutant, but not significantly relative to Col-0 (Fig. 3g). Together, these data indicate that camalexin plays a substantial role in the aphid resistance exhibited by Arabidopsis miRNA pathway mutants.

### Aphid fecundity is unaffected on JA and SA pathway mutants, but is increased on *ein2* plants

Our qRT-PCR data indicated that, in *dcl1* plants, the JA pathway transcript *LOX2* is induced following aphid infestation (Fig. 4b). This is in contrast with infested Col-0 and *dcl2*/*3*/*4*, where this transcript is not induced. This suggests that an aspect of JA signalling may be involved in miRNA mutant resistance. To assess this possibility, we exposed plants defective in JA signalling (*coi1*, *jar1*, 35S:*LOX2*) to aphids. Aphid fecundity was increased slightly on these lines relative to controls (Fig. S5); however, the increase was not statistically significant. This indicates that, in *dcl1* plants, there is differential regulation of the JA pathway relative to Col-0 and *dcl2*/*3*/*4*, but this has little bearing on the ability of these plants to resist aphid infestation. Aphid performance was also unchanged on plants deficient in SA signalling (Fig. S5).

As *dcl1* plants show increased induction of the ET-responsive *HEL* transcript following infestation (Figs 4e, S4), we investigated whether ET signalling affects aphid performance by assessing aphid performance on the ET-insensitive *etr1-1* and *ein2-5* mutants. Aphid fecundity was significantly higher on *ein2* plants relative to Col-0 (GLM, *P* < 0.05, *n* = 10) and was also higher on the *etr1* mutant, albeit not significantly, compared with Col-0 (Fig. 4f).

### Camalexin accumulation is increased in miRNA mutants

**Fig. 5:**
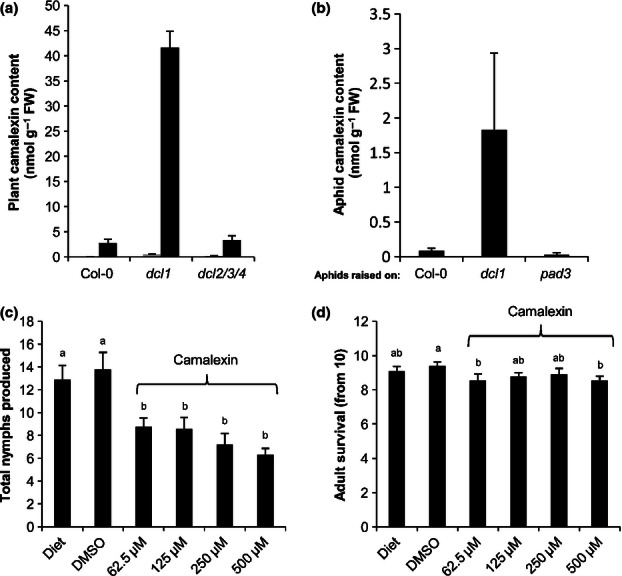
Camalexin accumulates in *dcl1* plants and *dcl1*-raised aphids and affects aphid reproductive development. (a) High-performance liquid chromatography (HPLC) (+MS) analysis of mock (grey bars) and aphid-infested (black bars) Col-0, *dcl1* and *dcl2*/*3*/*4* indicates that *dcl1* accumulates more camalexin when exposed to aphids. Bars represent mean camalexin content (± SE) from six biological replicates from two independent experiments. (b) Camalexin is detected in aphids and at higher levels in insects raised on *dcl1* than in Col-0- or *pad3*-raised aphids. Bars represent mean camalexin content (± SE) from six biological replicates from two independent experiments. (c, d) Feeding camalexin by artificial diet retards aphid fecundity (c), but has no effect on adult aphid survival (d). Dimethylsulfoxide (DMSO) (0.1%) served as a negative control. Each experiment contained five feeders at each condition. Bars represent the mean number of nymphs produced (c) or surviving adults (d) (± SE) from two independent experiments. Letters indicate differences at *P* < 0.05 as determined by *t* probabilities within a generalized linear model (GLM).

To assess whether increased *PAD3* expression in *dcl1* plants led to increased levels of camalexin, we exposed plants to 48 h of aphid infestation and measured camalexin content by HPLC and mass spectrometry (MS). We found that camalexin was present in similarly small quantities in Col-0, *dcl1* and *dcl2*/*3*/*4* plants without aphid challenge (Fig. 5a). However, on aphid exposure, there was increased camalexin accumulation in all plant genotypes, particularly in aphid-exposed *dcl1* compared with aphid-exposed Col-0 or *dcl2*/*3*/*4* (Fig. 5a). This result mirrors our previous data, which showed increased levels of *PAD3* mRNA in aphid-exposed *dcl1* plants relative to aphid-exposed Col-0 or *dcl2*/*3*/*4* (Figs 3a, S4). This indicates that elevated levels of *PAD3* expression correlate with increased camalexin accumulation during aphid attack.

### Camalexin is present in the phloem and is ingested by aphids during feeding

Camalexin is produced in significant quantities in aphid-challenged leaves; however, it is unknown whether camalexin is present in the Arabidopsis phloem stream and whether it is ingested by aphids on feeding. We obtained plants expressing a *PAD3p:GUS* transgene (Schuhegger *et al*., 2006) and exposed leaves to aphid infestation (Fig. S6). Leaves exposed to spores of the necrotrophic fungus *B. cinerea* showed GUS staining in a localized circular pattern surrounding the edge of the *B. cinerea* lesion (Kliebenstein *et al*., 2005; Fig. S6b). GUS staining was also observed in leaves exposed to GPA, although the pattern of staining differed considerably from that of *B. cinerea*-exposed leaves. The staining patterns on aphid-exposed leaves were much less uniform than those for *B. cinerea*, and were localized at aphid stylet penetration sites on the midveins of infested leaves (Fig. S6c–h). At the majority of feeding sites, GUS staining was observed in small patches around stylet penetrations (Fig. S6e,f). In a smaller proportion of feeding sites, stylet tracks were observed without any GUS staining (Fig. S6c,d), indicating that aphids had either abandoned probing, or had established a successful feeding site without activating a defence response involving *PAD3* induction. Third, on some leaves, GUS staining was observed in an extremely localized fashion (Fig. S6g,h), appearing to be confined to the vasculature tissue running perpendicular to the aphid feeding tracks. These data suggest that *PAD3* is expressed in the vasculature, and raises the possibility that camalexin is present in the phloem stream and is ingested by aphids when they feed.

To confirm that aphids ingest camalexin during feeding, we raised insects on plants considered to be high-camalexin-producing (*dcl1*), low-camalexin-producing (Col-0) and nonproducing (*pad3*). Aphids were harvested after 48 h of feeding and camalexin was quantified using the same methods as described for plant tissue samples. We were able to detect camalexin in aphids raised on all three plant genotypes (Fig. 5b), indicating that aphids are able to ingest this metabolite when feeding from Arabidopsis. In addition, we found that aphids raised on high-camalexin-producing hosts (*dcl1*) contained more camalexin than aphids raised on low-camalexin-producing hosts (Col-0; Fig. 5b). By contrast, there was little difference in the amount of camalexin detected in aphids raised on low-producing plants (Col-0) when compared with nonproducing plants (*pad3*; Fig. 5b). These data show that aphids ingest camalexin when feeding from Arabidopsis, and that a relationship exists between the quantity produced *in planta* and the quantity that accumulates in aphids.

### Camalexin inhibits adult aphid reproduction, but not survival

We next investigated the effects of supplying camalexin to aphids via an artificial diet. Ten adult aphids were transferred to parafilm sachet feeders containing a complex artificial diet used previously to examine aphid performance (Kim & Jander, 2007). Following 2 d of feeding, the numbers of remaining live adults were recorded as adult survival, and the total number of nymphs produced was recorded as fecundity. We found that, at all camalexin concentrations tested, fecundity was reduced significantly compared with both diet-only (Diet) and DMSO (0.1%) controls (GLM; *P* < 0.01, *n* = 10; Fig. 5c). By contrast, we found that adult survival was unchanged at all camalexin doses relative to the diet-only control (Fig. 5d). However, at camalexin doses of 62.5 and 500 μM, adult survival was significantly lower than that of the DMSO control (GLM; *P* < 0.05, *n* = 10; Fig. 5c). These data illustrate that camalexin can limit the number of individuals present within an aphid colony, predominantly through a deleterious effect on adult reproductive success.

### Aphid performance is partially restored on a *dcl1/pad3* double mutant

**Fig. 6:**
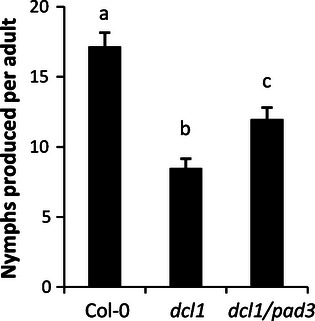
Aphid fecundity is partially restored on a *dcl1*/*pad3* double mutant. Aphid fecundity is higher on *dcl1*/*pad3* than on *dcl1* single mutants, but is not fully restored to wild-type levels. Bars represent the mean (± SE) of 18 plants of each genotype from three independent experiments. Letters indicate differences at *P* < 0.01 as determined by *t*-probabilities within a generalized linear model (GLM).

Finally, to confirm that *PAD3* and camalexin production are involved in the *dcl1* resistance phenotype, we introduced the *pad3* mutation into a *dcl1* genetic background. We isolated *dcl1*/*pad3* double mutants and tested aphid performance on these plants. We found that aphids reproduced significantly better on *dcl1*/*pad3* than on *dcl1* (GLM; *P* < 0.01, *n* = 18); however, fecundity was not fully restored to the levels observed on Col-0 plants (Fig. 6). This indicates that the camalexin pathway is responsible for a significant portion of the *dcl1* aphid-resistant phenotype.

## Discussion

In this study, we have shown that GPA produces significantly less progeny on Arabidopsis plants that aberrantly process miRNAs. Plants unable to process miRNAs respond to aphid infestation with increased induction of *PAD3* and production of camalexin. Aphids are more successful on the Arabidopsis *pad3* and *cyp79b2*/*cyp79b3* mutants defective in camalexin production. In addition, camalexin is present in the phloem stream and aphids raised on miRNA pathway mutants accumulate more camalexin than aphids raised on control plants. Aphids produce less progeny on artificial diets containing camalexin, indicating that this phytoalexin reduces the reproductive ability of GPA. Finally, aphid fecundity is partially restored for aphids raised on *dcl1*/*pad3* mutants relative to *dcl1*.

Our finding that aphids were less successful on *dcl1* plants was initially unexpected, as pathogen and insect performances have been shown to increase on silencing-deficient hosts (Deleris *et al*., 2006; Pandey & Baldwin, 2007). Indeed, type III secretion system (T3SS)-deficient *P. syringae* (which normally reproduces poorly on Arabidopsis) shows increased proliferation on Arabidopsis miRNA pathway mutants, but not on Arabidopsis plants defective in other silencing pathways (Navarro *et al*., 2008). Similarly, *Pseudomonas fluorescens* and *Escherichia coli*, which do not normally infect Arabidopsis, can multiply on Arabidopsis miRNA pathway mutants (Navarro *et al*., 2008). In addition, some RNA silencing mutants are hypersusceptible to infection by the vascular fungus *Verticillium* (Ellendorff *et al*., 2009). More specifically, for insects, an RDR1-silenced line of *Nicotiana attenuata* (irRdR1) is more susceptible to larvae of the solanaceous specialist *Manduca sexta* (Pandey & Baldwin, 2007). Nonetheless, there are several examples of increased resistance of Arabidopsis miRNA mutants to pathogens and pests. Both Arabidopsis miRNA and siRNA pathway mutants exhibit increased resistance to the cyst nematode *Heterodera schachtii* (Hewezi *et al*., 2008), and *dcl1* plants are resistant to tumour formation following stab inoculation with tumorigenic *Agrobacterium* (Dunoyer *et al*., 2006). This may be expected, as miRNAs are integral players in plant development, and cyst nematodes and *Agrobacterium* reprogramme plant development to generate cysts and galls, respectively, which provide feeding and replication sites for these plant colonizers. Thus, our observation that aphids do less well on Arabidopsis miRNA mutants may be a consequence of the highly specialized feeding mode of aphids. GPA does not form noticeable galls, but may still need to modulate specific developmental or basic plant defence processes that are regulated by miRNAs in order to establish long-term feeding sites. The salivary components that aphids release into cells whilst they navigate to the phloem and during phloem feeding (Will *et al*., 2007; Mutti *et al*., 2008; De Vos & Jander, 2009; Bos *et al*., 2010; Pitino & Hogenhout, 2013) may induce these modulations. We propose that the GPA colonization efficiency of Arabidopsis is enhanced by the ability of this aphid to modulate specific plant processes that are regulated by miRNAs.

*dcl1* plants display greater resistance to GPA infestation, and our data suggest that this is a result, in part, of the hyperactivation of the camalexin defence pathway. By contrast, this pathway is only modestly induced in aphid-susceptible Col-0 and *dcl2*/*3*/*4* plants. One possibility is that factors that act as brakes or suppressors of defence hyperactivation in Col-0 or *dcl2*/*3*/*4* are ineffective or absent in *dcl1* plants. Suppressors of hyperactivation may be protein effectors present in aphid saliva that can modify aspects of host physiology and suppress defensive mechanisms. Therefore, host proteins involved in camalexin production or specific miRNAs involved in the management of this pathway may be targets for as yet uncharacterized aphid salivary effectors. Indeed, effectors from a plant pathogen are capable of interfering with host miRNA processing (Navarro *et al*., 2008). Another possibility is that plants actively manage their response through the induction of specific miRNA species that target transcripts involved in the camalexin pathway. This control mechanism would be largely disabled in *dcl1* plants. As large quantities of camalexin are toxic to Arabidopsis cells in culture (Rogers *et al*., 1996), this dampening effect may represent a form of plant self-defence.

In Arabidopsis, some miRNAs target transcripts related to secondary metabolism. One group of miRNAs (miR160, miR167, miR390, miR393) is specifically related to auxin signalling (Zhang *et al*., 2011), which is linked to camalexin and glucosinolate biosynthesis. In addition, miR393 has a role in the plant immune response as it is induced following exposure to the PAMP flg22 (Navarro *et al*., 2006; Li *et al*., 2010), and following inoculation of both virulent and avirulent strains of *P. syringae* pv. *tomato* (*Pst*; Zhang *et al*., 2011). It has also been reported that miR393 has a role in resource allocation between the glucosinolate and camalexin pathways (Robert-Seilaniantz *et al*., 2011).

Aphids transmit one-third of *c*. 800 described plant viruses (Ng & Perry, 2004; Hogenhout *et al*., 2008). Many of these viruses encode suppressor molecules which block antiviral RNA silencing (Ding & Voinnet, 2007) and can interfere with the miRNA pathway during infection (Chapman *et al*., 2004). Silencing suppression is crucial to promote virus infectivity; however, suppression of the miRNA pathway might have a negative impact on the fecundity of the aphid vectors through the mechanisms described here. The relationship between virus and insect will strongly determine the outcome of this tritrophic interaction. Viruses that are acquired rapidly and transmitted by aphids will benefit from plant behaviour that discourages aphid settling (Mauck *et al*., 2010). By contrast, viruses that require longer acquisition times, such as those that are phloem limited, may act to extend aphid feeding time at a particular feeding site (Eigenbrode *et al*., 2002).

Our qRT-PCR assays indicated that aphid-resistant *dcl1* plants increase transcription of an ET-responsive gene relative to susceptible Col-0 and *dcl2*/*3*/*4* plants following aphid colonization. Fecundity assays confirmed the involvement of ET signalling in resistance, as aphid performance was improved significantly on *ein2* mutants. Our result, showing no change in aphid fecundity on *etr1*, is consistent with previous studies in which the performances of GPA and *Brevicoryne brassicae* were either unaffected or reduced on *etr1* mutants (Mewis *et al*., 2005, 2006). Other laboratories have demonstrated that saliva-induced aphid resistance is independent of EIN2 and ET signalling (De Vos & Jander, 2009), whereas EIN2 is known to be critical for resistance to GPA following treatment with the bacterial protein harpin (Dong *et al*., 2004; Liu *et al*., 2011). It remains a possibility that altered regulation of this signalling mechanism contributes to the *dcl1* resistance phenotype.

Aphid fecundity was increased on the *pad3* and *cyp79b2*/*cyp79b3* mutants relative to Col-0. By contrast, aphid performance was unchanged on the *cyp81f2* mutant. Taken together, these results indicate that, under our experimental conditions, the production of camalexin is a major resistance factor. This is in contrast with the observations of Pegadaraju *et al*. (2005), who found no statistically significant increase in GPA colonization ability on *pad3* mutants. In addition, Kim *et al*. (2008) found no change in fecundity of aphids raised on *cyp79b2*/*cyp79b3* mutants relative to wild-type plants. However, in both cases, nonaged aphids were exposed to the mutant plants for a relatively short period, that is 2–5 d, whereas, in the experiments reported herein, the nymphs were born on the mutant plants and reared on these plants to adulthood (*c*. 16 d), during the course of which they began to produce nymphs themselves. Thus, differences in the experimental procedures may account for the different outcomes. Indeed, the *dcl1* resistance phenotype was absent when experiments were carried out following a previously published protocol (Pegadaraju *et al*., 2005; Fig. S7). It is also possible that the aphid colonies maintained by different laboratories have varying susceptibilities to different phytochemicals. Our results are in agreement with those of Kusnierczyk *et al*. (2008), who found that *B. brassicae* (cabbage aphid) is more successful on *pad3* relative to wild-type Arabidopsis when both plants are pretreated with UV light to induce camalexin production. In these experiments, aged nymphs were raised on test plants for 13 d, a protocol very similar to our own assay. Furthermore, aphids produce less progeny on artificial diets containing camalexin compared with control diets, confirming that camalexin has a negative impact on GPA performance. This indicates an unsuspected depth to camalexin function beyond antifungal and antibacterial defence. This work also highlights the extensive role of the miRNA-mediated regulation of secondary metabolic defence pathways with relevance to resistance against an aphid pest.

## Supporting Information

**Fig. S1** Phenotypes of all plants used in the fecundity screen of the silencing mutants.

**Fig. S2** Investigation into the temporal response of Col-0 plants to *Myzus persicae* (green peach aphid, GPA) infestation.

**Fig. S3** Induction of the pathogen response-related gene *MITOGEN-ACTIVATED PROTEIN KINASE3* (MPK3) is highest in *dcl1* plants.

**Fig. S4** Expression levels of *PHYTOALEXIN DEFICIENT3 (PAD3)* and *HEVEIN-LIKE (HEL)* at 24 and 48 h post-inoculation (hpi) in Col-0, *dcl1* and *dcl2/3/4* plants.

**Fig. S5** Aphid fecundity trials on Arabidopsis salicylic acid (SA) and jasmonic acid (JA) pathway mutants.

**Fig. S6** Spatial induction of a *CYP71B15p::GUS (PAD3p::GUS)* transgene following aphid feeding.

**Fig. S7** Aphid fecundity trial on Arabidopsis Col-0, *dcl1* and *dcl2/3/4* using a previously described fecundity/survival assay protocol.

**Table S1** Oligonucleotide primers used for quantitative real-time polymerase chain reaction (qRT-PCR) experiments
