# Supplementary material for: Resistance of Arabidopsis thaliana to the green peach aphid, Myzus persicae, involves camalexin and is regulated by microRNAs
Source: New Phytol. 2013 Mar 25;198(4):1178–90. doi: 10.1111/nph.12218 (PMC3666093; doi:10.1111/nph.12218)
Supplement: Supplementary file 1 [file nph0198-1178-SD1.pptx]

## Slide 1
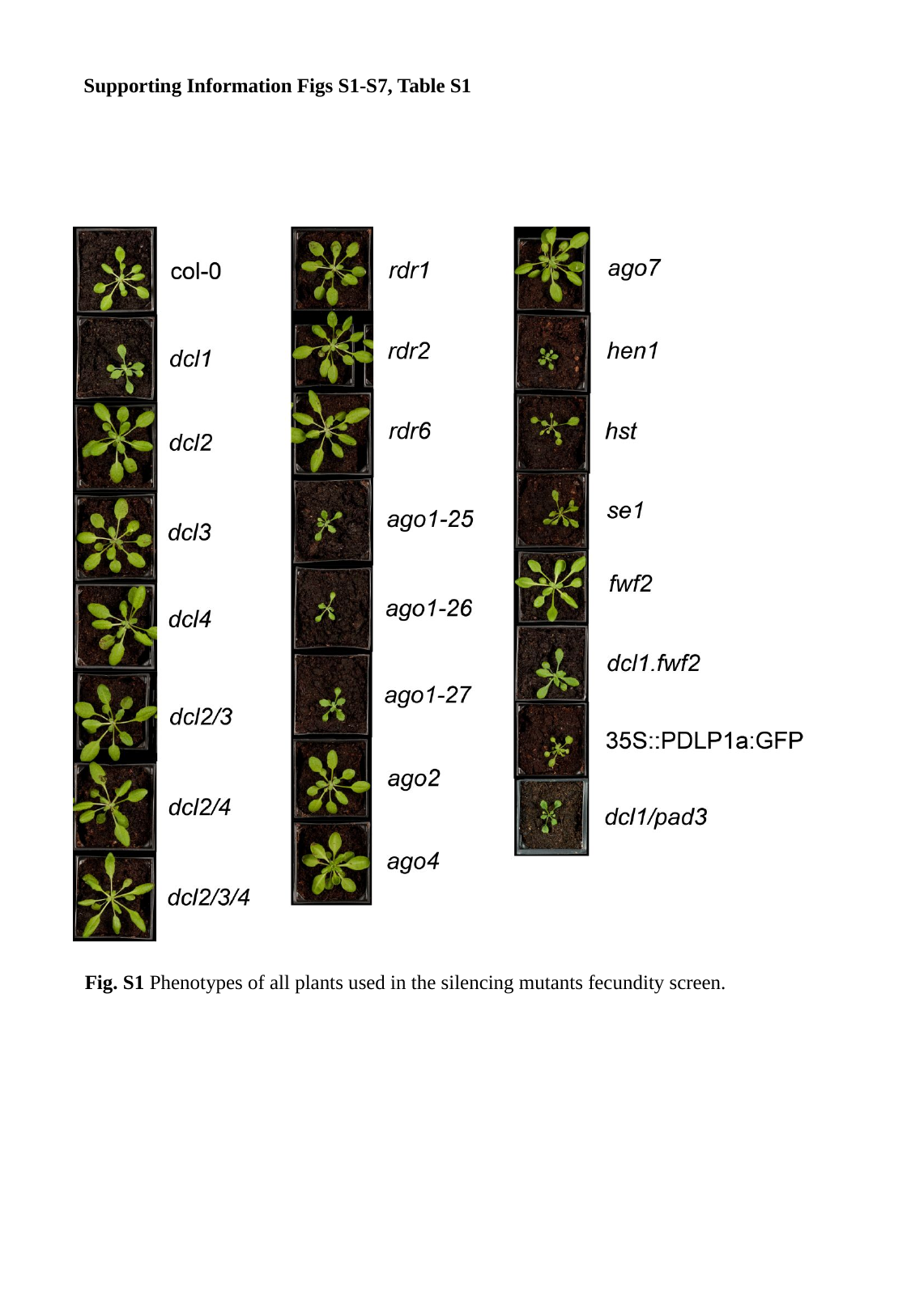

Supporting Information Figs S1-S7, Table S1
Fig. S1 Phenotypes of all plants used in the silencing mutants fecundity screen.

## Slide 2
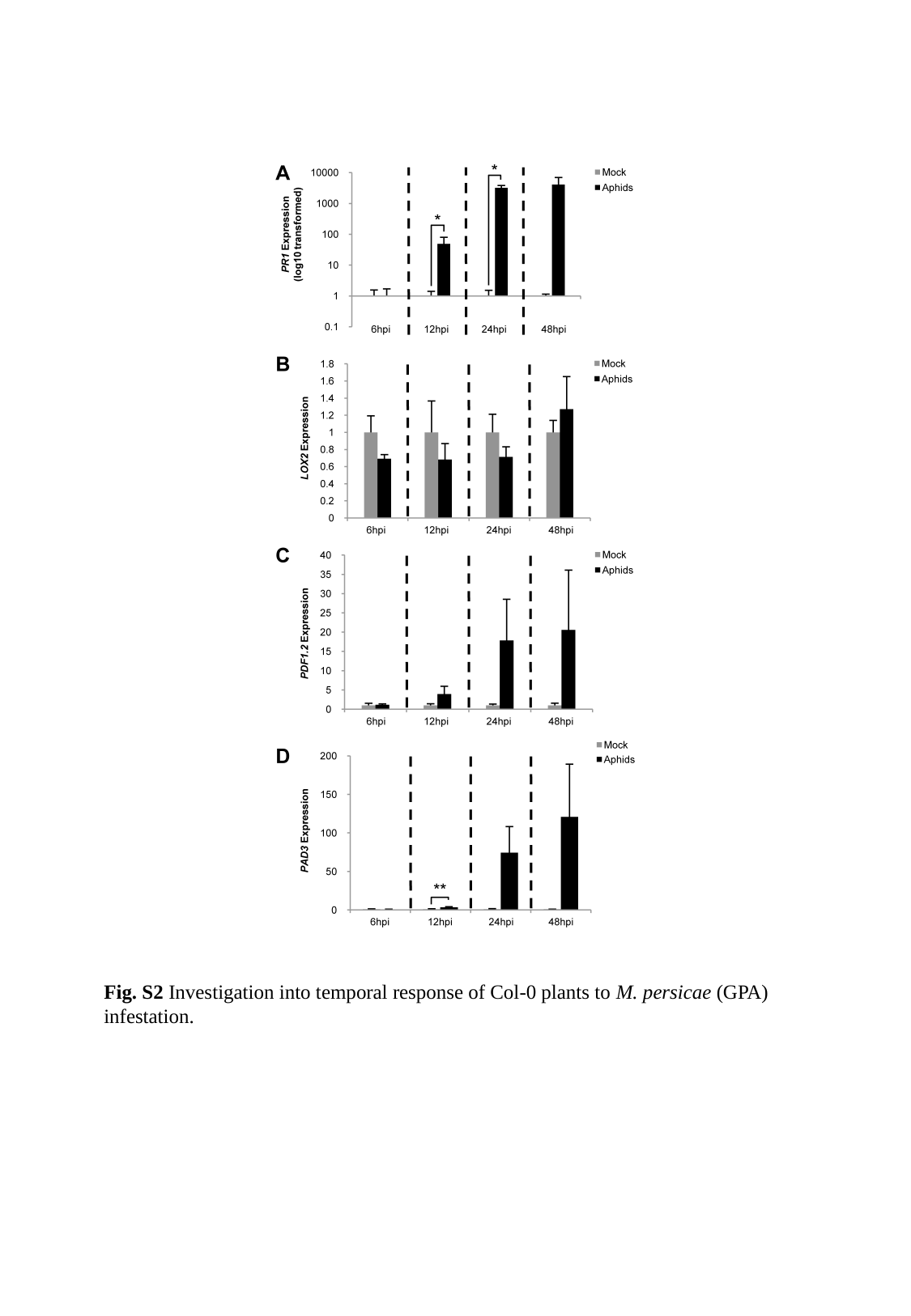

Fig. S2 Investigation into temporal response of Col-0 plants to M. persicae (GPA) infestation.

## Slide 3
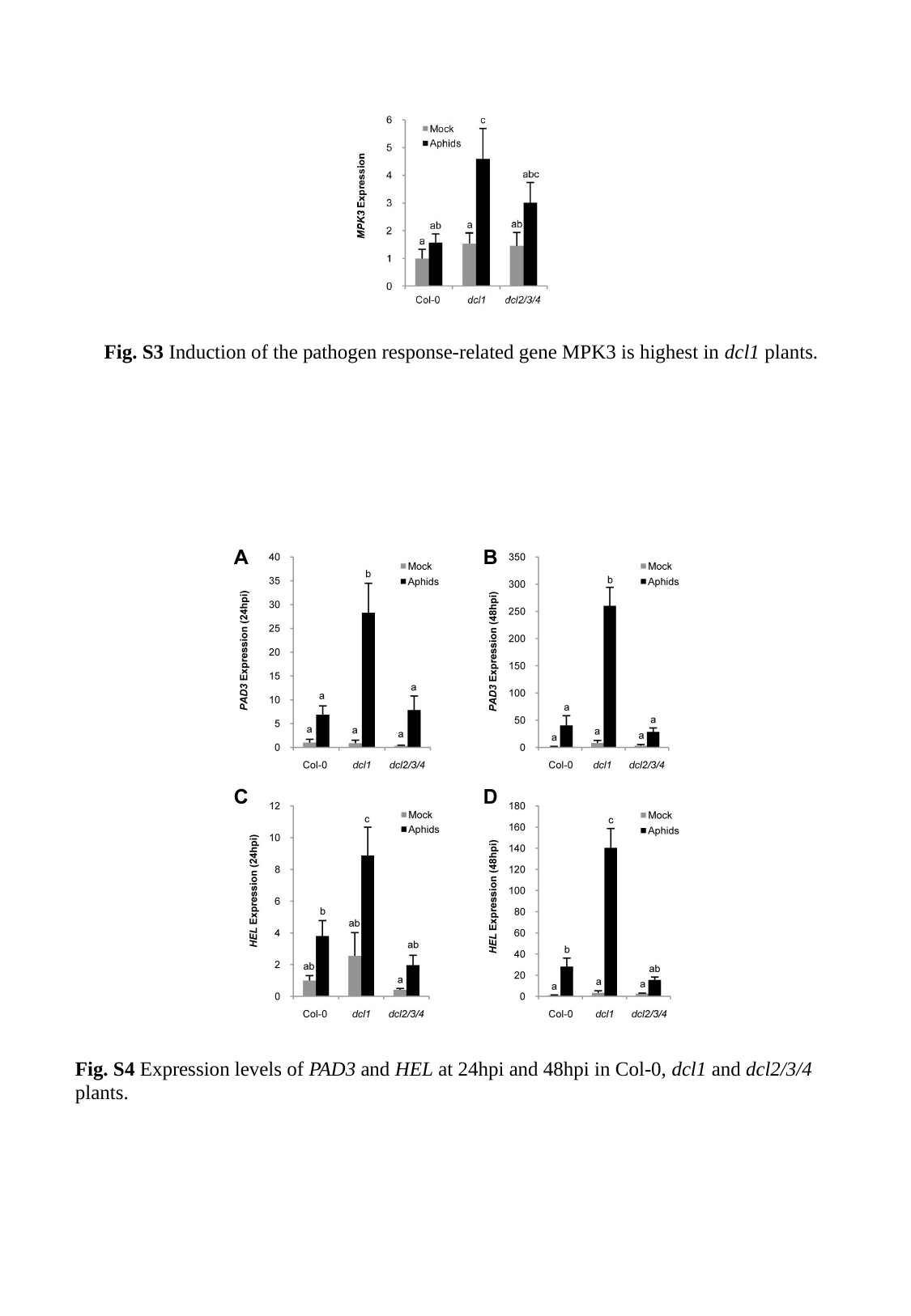

Fig. S3 Induction of the pathogen response-related gene MPK3 is highest in dcl1 plants.
Fig. S4 Expression levels of PAD3 and HEL at 24hpi and 48hpi in Col-0, dcl1 and dcl2/3/4 plants.

## Slide 4
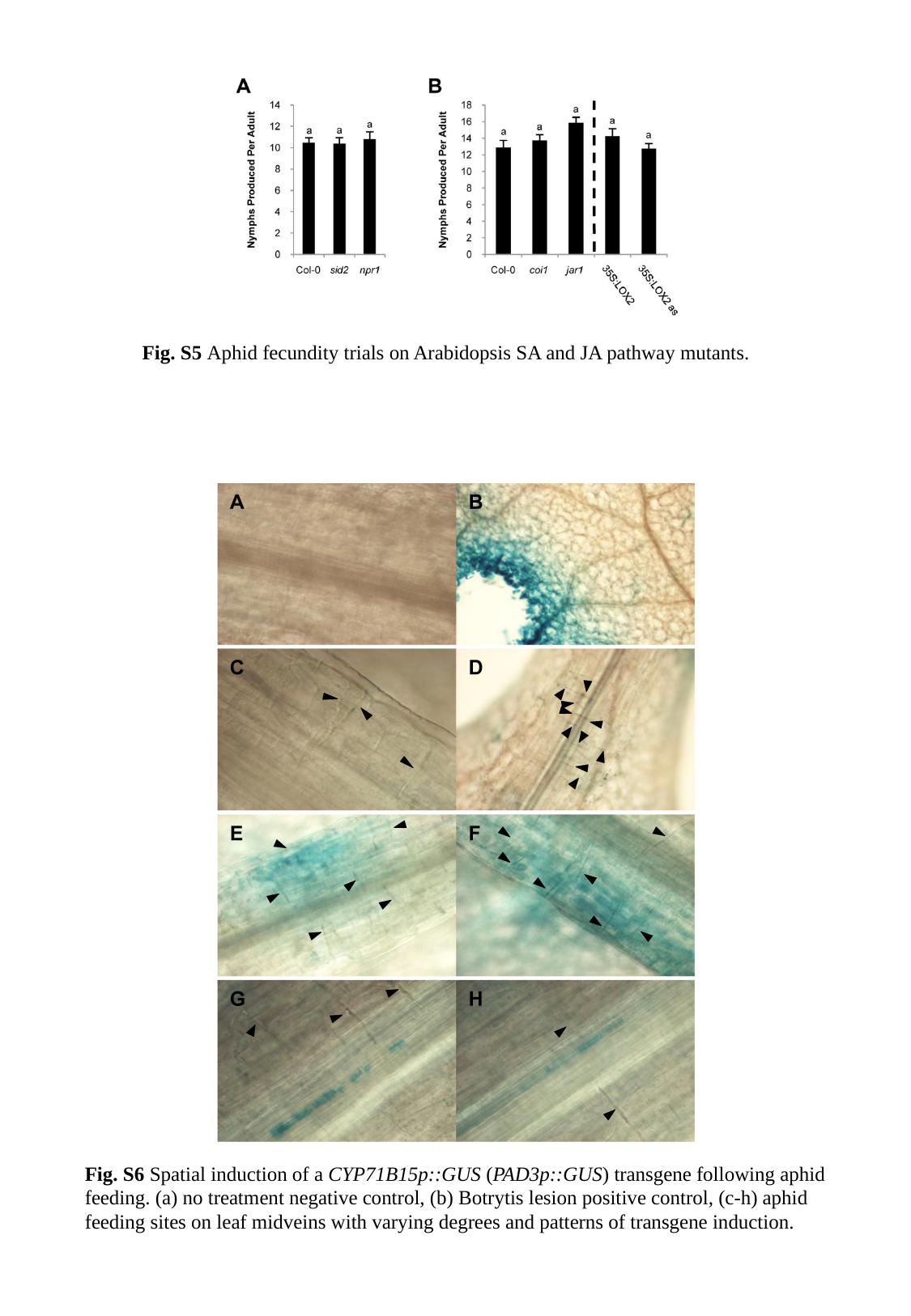

Fig. S5 Aphid fecundity trials on Arabidopsis SA and JA pathway mutants.
Fig. S6 Spatial induction of a CYP71B15p::GUS (PAD3p::GUS) transgene following aphid feeding. (a) no treatment negative control, (b) Botrytis lesion positive control, (c-h) aphid feeding sites on leaf midveins with varying degrees and patterns of transgene induction.

## Slide 5
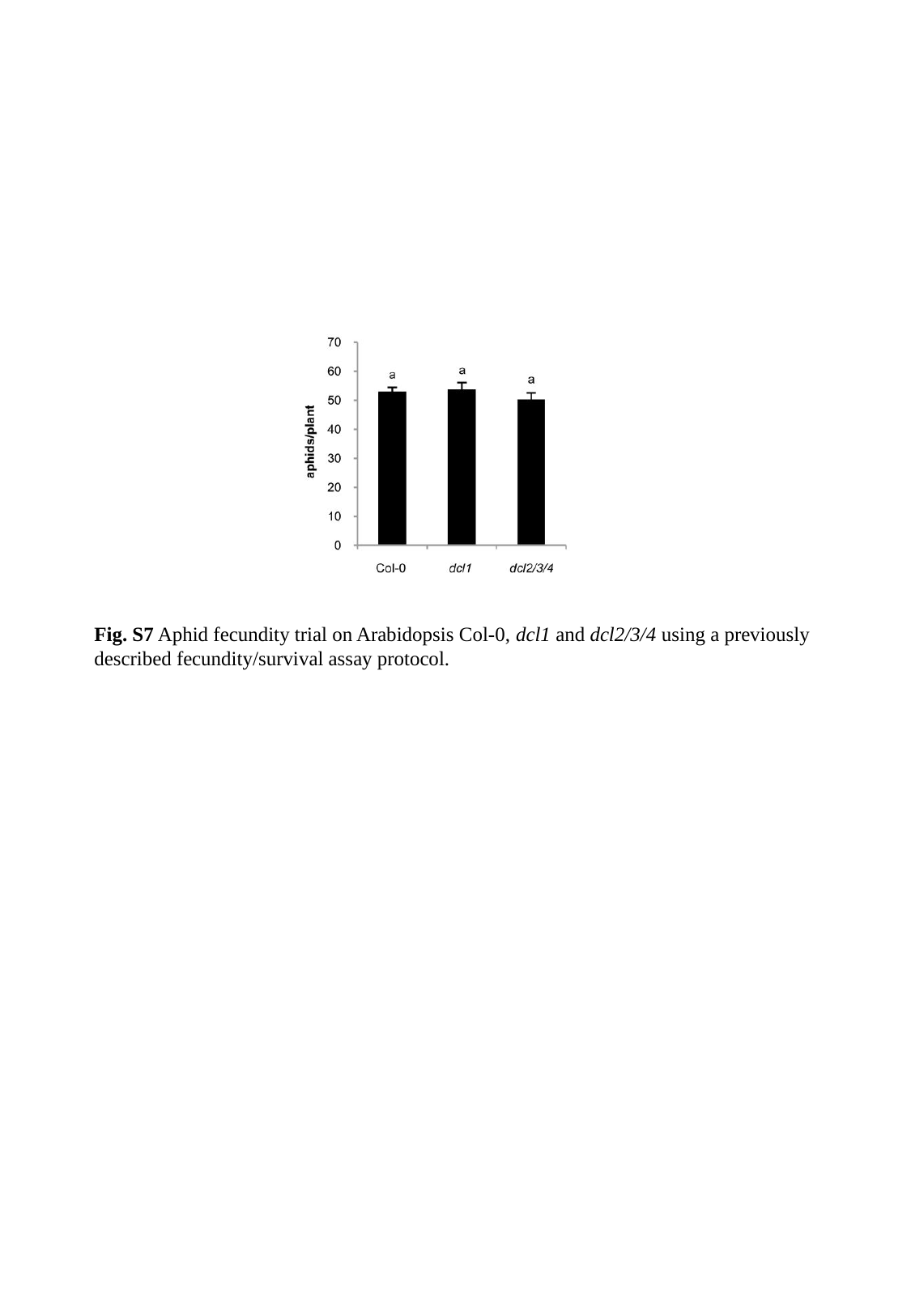

Fig. S7 Aphid fecundity trial on Arabidopsis Col-0, dcl1 and dcl2/3/4 using a previously described fecundity/survival assay protocol.

## Slide 6
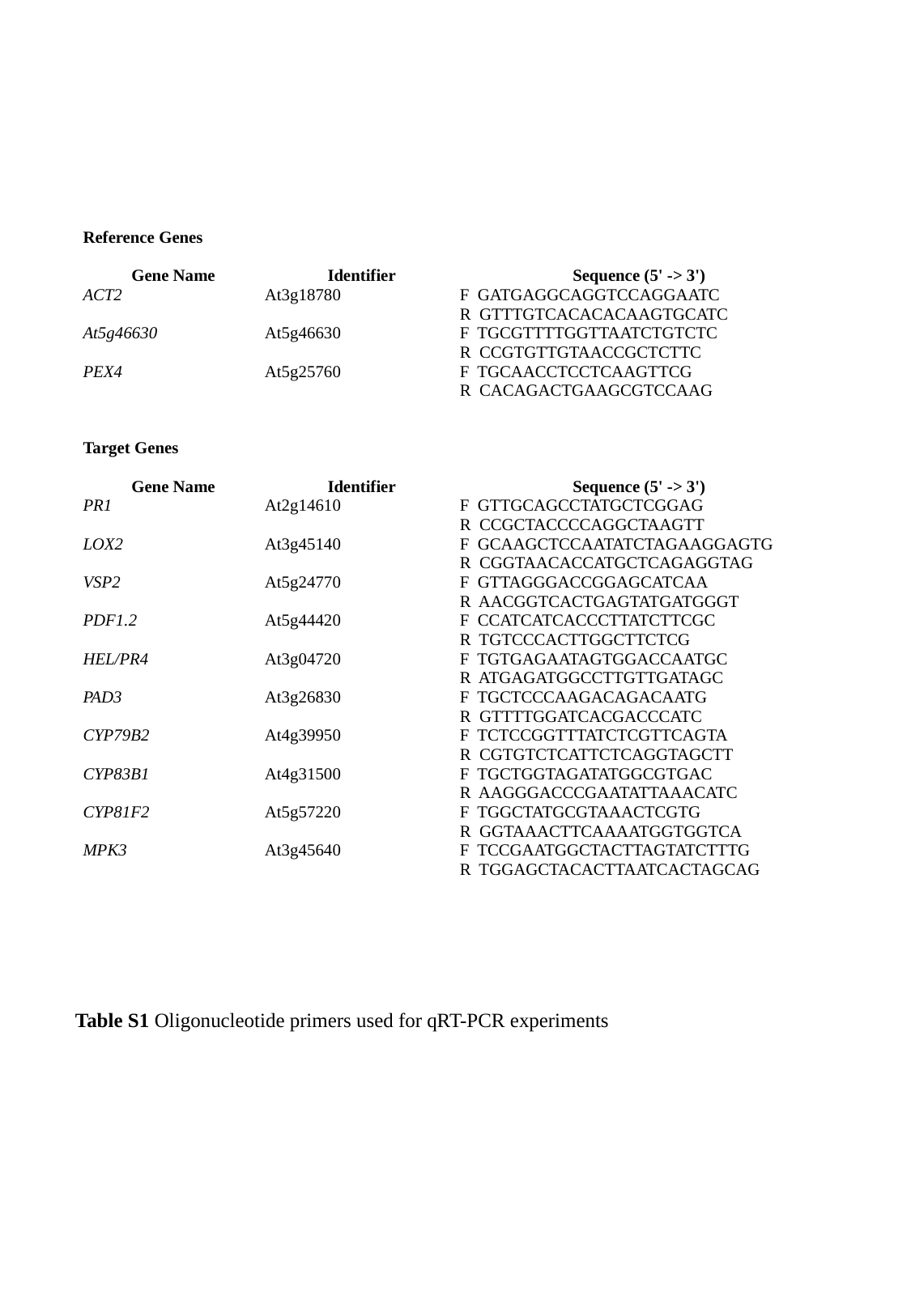

| | | |
| --- | --- | --- |
| Reference Genes | | |
| | | |
| Gene Name | Identifier | Sequence (5' -> 3') |
| ACT2 | At3g18780 | F GATGAGGCAGGTCCAGGAATC |
| | | R GTTTGTCACACACAAGTGCATC |
| At5g46630 | At5g46630 | F TGCGTTTTGGTTAATCTGTCTC |
| | | R CCGTGTTGTAACCGCTCTTC |
| PEX4 | At5g25760 | F TGCAACCTCCTCAAGTTCG |
| | | R CACAGACTGAAGCGTCCAAG |
| | | |
| | | |
| Target Genes | | |
| | | |
| Gene Name | Identifier | Sequence (5' -> 3') |
| PR1 | At2g14610 | F GTTGCAGCCTATGCTCGGAG |
| | | R CCGCTACCCCAGGCTAAGTT |
| LOX2 | At3g45140 | F GCAAGCTCCAATATCTAGAAGGAGTG |
| | | R CGGTAACACCATGCTCAGAGGTAG |
| VSP2 | At5g24770 | F GTTAGGGACCGGAGCATCAA |
| | | R AACGGTCACTGAGTATGATGGGT |
| PDF1.2 | At5g44420 | F CCATCATCACCCTTATCTTCGC |
| | | R TGTCCCACTTGGCTTCTCG |
| HEL/PR4 | At3g04720 | F TGTGAGAATAGTGGACCAATGC |
| | | R ATGAGATGGCCTTGTTGATAGC |
| PAD3 | At3g26830 | F TGCTCCCAAGACAGACAATG |
| | | R GTTTTGGATCACGACCCATC |
| CYP79B2 | At4g39950 | F TCTCCGGTTTATCTCGTTCAGTA |
| | | R CGTGTCTCATTCTCAGGTAGCTT |
| CYP83B1 | At4g31500 | F TGCTGGTAGATATGGCGTGAC |
| | | R AAGGGACCCGAATATTAAACATC |
| CYP81F2 | At5g57220 | F TGGCTATGCGTAAACTCGTG |
| | | R GGTAAACTTCAAAATGGTGGTCA |
| MPK3 | At3g45640 | F TCCGAATGGCTACTTAGTATCTTTG |
| | | R TGGAGCTACACTTAATCACTAGCAG |
| | | |
Table S1 Oligonucleotide primers used for qRT-PCR experiments
